# PCGIMA: developing the web server for human position-defined CpG islands methylation analysis

**DOI:** 10.3389/fgene.2024.1367731

**Published:** 2024-03-13

**Authors:** Ming Xiao, Yi Xiao, Jun Yu, Le Zhang

**Affiliations:** ^1^ College of Computer Science, Sichuan University, Chengdu, China; ^2^ Tianfu Engineering-oriented Numerical Simulation and Software Innovation Center, Chengdu, China; ^3^ CAS Key Laboratory of Genome Sciences and Information, Chinese Academy of Sciences, Beijing Institute of Genomics, Beijing, China; ^4^ University of Chinese Academy of Sciences, Beijing, China; ^5^ Key Laboratory of Systems Biology, Chinese Academy of Sciences, Hangzhou Institute for Advanced Study, University of Chinese Academy of Sciences, Hangzhou, China; ^6^ Key Laboratory of Systems Health Science of Zhejiang Province, Hangzhou Institute for Advanced Study, University of Chinese Academy of Sciences, Hangzhou, China

**Keywords:** position-defined CGIs, DNA methylation, genome annotation, high performance computing, genome analysis

## Abstract

**Introduction:** CpG island (CGI) methylation is one of the key epigenomic mechanisms for gene expression regulation and chromosomal integrity. However, classical CGI prediction methods are neither easy to locate those short and position-sensitive CGIs (CpG islets), nor investigate genetic and expression pattern for CGIs under different CpG position- and interval- sensitive parameters in a genome-wide perspective. Therefore, it is urgent for us to develop such a bioinformatic algorithm that not only can locate CpG islets, but also provide CGI methylation site annotation and functional analysis to investigate the regulatory mechanisms for CGI methylation.

**Methods:** This study develops Human position-defined CGI prediction method to locate CpG islets using high performance computing, and then builds up a novel human genome annotation and analysis method to investigate the connections among CGI, gene expression and methylation. Finally, we integrate these functions into PCGIMA to provide relevant online computing and visualization service.

**Results:** The main results include: (1) Human position-defined CGI prediction method is more efficient to predict position-defined CGIs with multiple consecutive (d) values and locate more potential short CGIs than previous CGI prediction methods. (2) Our annotation and analysis method not only can investigate the connections between position-defined CGI methylation and gene expression specificity from a genome-wide perspective, but also can analysis the potential association of position-defined CGIs with gene functions. (3) PCGIMA (http://www.combio-lezhang.online/pcgima/home.html) provides an easy-to-use analysis and visualization platform for human CGI prediction and methylation.

**Discussion:** This study not only develops Human position-defined CGI prediction method to locate short and position-sensitive CGIs (CpG islets) using high performance computing to construct MR-CpGCluster algorithm, but also a novel human genome annotation and analysis method to investigate the connections among CGI, gene expression and methylation. Finally, we integrate them into PCGIMA for online computing and visualization.

## 1 Introduction

CpG island (CGI) methylation is one of the key epigenomic mechanisms for gene expression regulation and chromosomal integrity (Dor and Cedar, 2018). Especially, recent studies have reported that position-sensitive CGI co-methylation mechanism is essential for such functions that are related to histone modification (Ming, et al., 2021). However, it is neither easy for current commonly used classical CGI island prediction methods (Gardinergarden and Frommer, 1987; Han et al., 2008; Takahashi et al., 2017) to locate those short and position-sensitive CGIs which called CpG islets (Hackenberg et al., 2006) due to the length limitation, nor investigate relationship among CGI density, methylation, and gene expression specificity. Therefore, it is urgent for us to develop such a bioinformatic algorithm that not only can locate short and position-sensitive CGIs (CpG islets), but also provide CGI methylation site annotation and functional analysis to investigate the regulatory mechanisms for CGI methylation (http://www.combio-lezhang.online/pcgima/home.html).

For CGI perdition method, we usually employ the unsupervised clustering methods such as CpGCluster (Hackenberg et al., 2006) and CPG_MI (Su et al., 2009) to locate CGIs with shorter length than the supervised (Bock et al., 2007; Ning et al., 2017), since these unsupervised algorithms do not need consider the constraints of CGI length and content ratio (Hackenberg et al., 2010). However, these methods are not only time-consuming for the big dataset, but also cannot investigate the genetic characteristics of CGIs under different CpG interval parameters. Therefore, our first scientific question is how to develop a novel CGI prediction method with CpG interval parameters selective feature and high-performance computing, and investigate the differences in genetic characteristics such as CpG coverage, CGI length, and GC content of CGIs under various CpG interval parameters.

Several previous studies have interrogated the connections between methylation and CGI (Reik, 2007; Smith et al., 2012; Liu et al., 2016; El-Maarri, 2019; Acton et al., 2021). For example, Ziller et al. (2013) have turned out that not only the hypermethylation of promoter CGI is related to gene expression, but also CGI methylation in the gene body is positively correlated with gene expression. However, these studies usually interrogate the methylation characteristics of CGI from partial sequence regions rather than genome-wide perspective. Meanwhile, although our previous studies (Zhang et al., 2018; Zhang et al., 2021a) have analyzed the relationship between CGI density and gene expression after annotating genome-wide CGI-related genes (CGI+) into high-CGI (HCGI), intermediate-CGI (ICGI), and low-CGI (LCGI) genes based on the classification of CGI density (Weber et al., 2007; Zhu et al., 2008), we are still unclear the relationship between CpG methylation and gene expression. Thus, our second scientific question is how to build up a human genome-wide CGI-based methylation and gene expression annotation and analysis method to investigate the relationship among CGI density, methylation, and gene expression specificity.

Meanwhile, although several CpG methylation online service are already available (Raney et al., 2010; Di et al., 2018; Xiong et al., 2019), most of them only focus on CpG island prediction and data downloading, but not provide visualization and analysis for the distribution of CGI in different sequence regions and the connections between methylation status of CGIs and gene expression. Therefore, our third scientific question is how to establish an easy-to-use web service for fast CGIs prediction and visualization of the connections between CGIs and methylation.

For these reasons, we propose three major innovations to answer the above scientific questions.

Firstly, we develop an unsupervised clustering-based CGI prediction method (Human position-defined CGI prediction), which not only employs high performance computing technology to accelerate its predictive speed, but also offers a parameter selective option that can help us to locate short CGIs (position-defined CGIs) with unique location- or sequence-sensitive features and explore the differences in the genetic characteristics of CGIs under various CpG interval parameters.

Secondly, we build up a novel human genome annotation and analysis function (Human position-defined CGI annotation and analysis), which not only can study the methylation characteristics of CGIs from a genome-wide perspective by computing the methylation level of all CpG sites in the human genome, but also improve the previous CpG-Island-based human gene expression annotation and analysis method (Zhang et al., 2021a) by integrating genome-wide methylation annotation to further investigate the connections among CGI density, gene expression and methylation.

Thirdly, we establish an easy-to-use web service “Position-defined CGI methylation analysis (PCGIMA)” with relevant CGI prediction, annotation, and data analysis functions, which provides us an online platform for further study on the regulation mechanism of CGI and methylation.

In conclusion, we develop a bioinformatic algorithm and web service to investigate the regulatory mechanism of CGI methylation. The main results include: 1) Human position-defined CGI prediction method is more efficient to predict position-defined CGIs with multiple consecutive (d) values and locate more potential short CGIs than previous CGI prediction methods; 2) Our annotation and analysis method not only can investigate the connections between position-defined CGI methylation and gene expression specificity from a genome-wide perspective, but also can analyze the potential association of position-defined CGIs with gene functions; (3) PCGIMA provides an easy-to-use analysis and visualization platform for human CGI prediction and methylation.

## 2 Materials and methods

**FIGURE 1:**
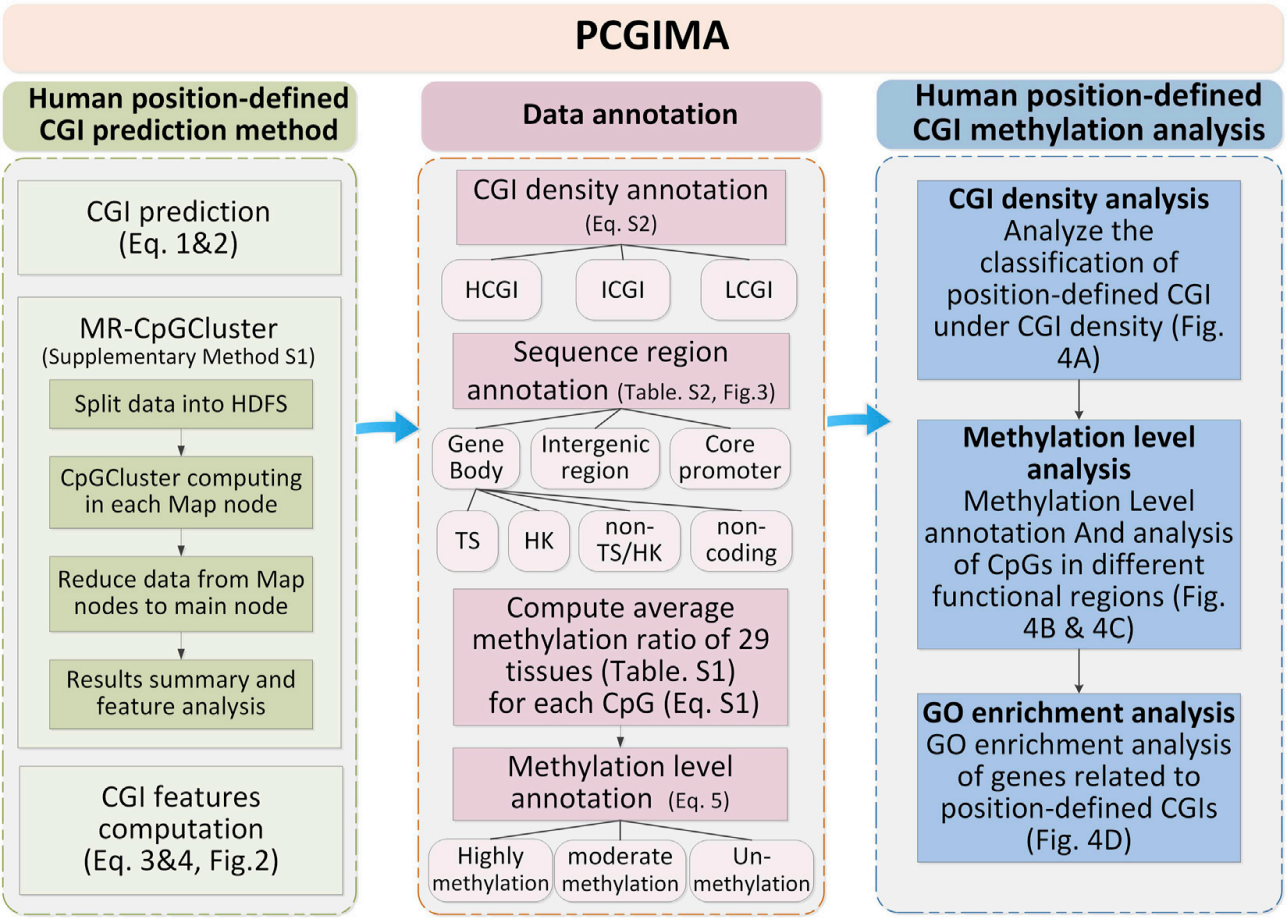
Workflow of the study.

This study downloads human genome data from GRCh38 assembly (Schneider et al., 2017) at NCBI (Pruitt et al., 2005). To classify CGIs into density-defined and position-defined groups, we download human CGIs data and annotations from UCSC (Casper et al., 2018). Next, we use human genome annotated data (release 24) in GenBank GBFF format (Clark et al., 2016) from GENCODE (Wright et al., 2016) to define different sequence regions. Finally, to study the methylation level of CpG sites in different sequence regions, we obtain all CpG methylation data of 29 human tissues (Supplementary Table S1), including heart, spleen, lung and esophagus, from ENCODE databases (Harrow et al., 2006). In order to ensure data consistency, the above-listed annotation and methylation data are all annotated according to GRCh38 (Schneider et al., 2017). Figure 1 describes the workflow of the study with three essential steps: Human position-defined CGI prediction (left side of Figure 1), Data annotation (right side of Figure 1), and Human position-defined CGI methylation analysis (Bottom side of Figure 1).

Here, we describe the key equations as follows:

(1) CGI prediction: We employed Eq. 1 to define CpGs clusters (Hackenberg et al., 2006) at the start. Next, we consider these CpG clusters with small p-values (Eq. 2) as CGIs (Hackenberg et al., 2006).

$$d_{i}=x_{i+1}-x_{i}-1$$ (1)

Here, x and I represent the position and index of a CpG, respectively.

$$P\left(d\right)={\left(1-p\right)}^{d-1}p$$ (2)

P(d) represents the probability to find a distance d between neighboring CpGs. p corresponds to the probability of CpGs in the sequence. Since our previous studies (Zhang et al., 2018; Zhang et al., 2021a) has led to a conclusion that LAUPs (Lineage-associated underrepresented permutations) are closely related to CGIs and the shortest LAUPs of mammals range from 10bp to 14bp in length, here we use the intermediate value of d = 12bp.

(2) MR-CpGCluster: We develop a MR-CpGCluster algorithm (Supplementary Figure S1) to speed up CGI predict procedure based on MapReduce (Dittrich and Quiané-Ruiz, 2012) and Hadoop Streaming (Dede et al., 2016) techniques detailed by Supplementary Method S1 for Human position-defined CGI prediction method. Finally, our method computes the CGI features of the position-defined CGIs for subsequent analysis.
(3) CGI features computation: To compare the CGIs under different CpG distance intervals (Eq. 1), we compute CGI length, CG content, CpG O/E ratio (Gardinergarden and Frommer, 1987) (Eq. 3) and CpG density (Eq. 4) for each CGI (Hackenberg et al., 2006).

$$O/E=\frac{\mathrm{CpNum}}{\mathrm{CNum}\times \mathrm{GNum}}\times N$$ (3)

$$\mathrm{CpGdensity}=\frac{\mathrm{CpNum}}{N}$$ (4)

Here, N is the length of the CGI, CpGNum, CNum and GNum represent the number of CpG, number of C, number of G respectively.

(4) Methylation level annotation: Eq. 5 classifies methylation ratio into three levels with respect to the definition (Ziller et al., 2013).

$$\mathrm{Methylation level}\left(chr,p\right)=\left\{\begin{array}{cc} 1,\mathrm{highly mehylated} & \mathrm{methylation}\_\mathrm{ratio}\left(chr,p\right)>0.75\\ 2,\mathrm{unmethylated} & \mathrm{methylation}\_\mathrm{ratio}\left(chr,p\right)<0.1\\ 3,\mathrm{moderate methylated} & \mathrm{otherwise} \end{array}\right.$$ (5)

Here, chr and p represent the chromosome and position of a CpG site, respectively.

## 3 Results

### 3.1 Human position-defined CGI prediction method

**FIGURE 2:**
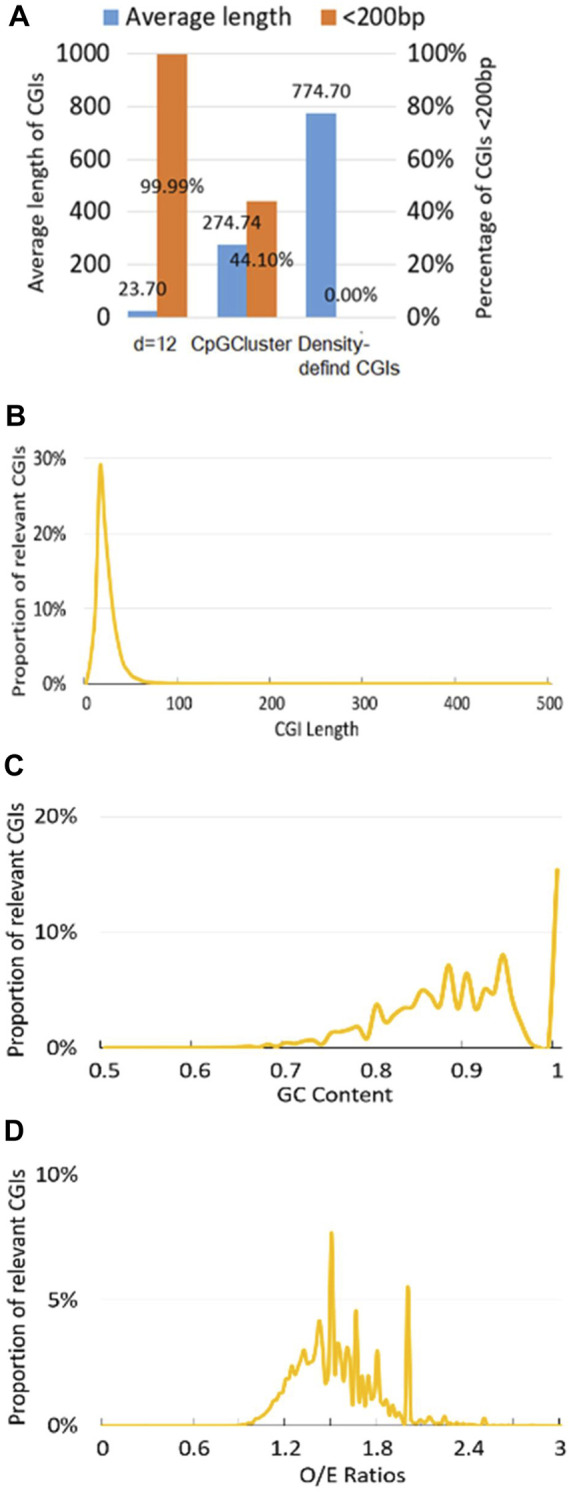
Position-defined CGI prediction and analysis. **(A)** CGIs comparative analysis. The proportion distribution of CGI at different **(B)** CGI length, **(C)** GC content, and **(D)** CpG O/E ratios (Eq. 3) under CpG interval d = 12bp.

Indicated by previous study (Hackenberg et al., 2006), we consider CGIs as potentially functionally short islands (CpG islets) if length of CGIs is less than 200bp. Here, Figure 2A demonstrates that Human position-defined CGI prediction method not only can locate the shortest average length (23.7bp) under CpG interval d = 12bp, but also the percentage of CGIs <200bp for Human position-defined CGI prediction method are greater than both CpGCluster method (Hackenberg et al., 2006) and density-defined CGI prediction method (Weber et al., 2007; Zhang et al., 2021a).

Also, since proportion distribution of CGI features is closely related to the regulatory mechanisms for CGI methylation (Hackenberg et al., 2010), Human position-defined CGI prediction method can describe the proportional distribution of the predicted CGIs at different CGI length (Figure 2B), GC content (Figure 2C), and O/E (Figure 2D). Here, we employ default setup for CpG interval, d = 12bp (Zhang et al., 2018; Zhang et al., 2021a).

It should be noted that Human position-defined CGI prediction method can parallel carry out position-defined CGI prediction and comparative analysis for multiple CpG intervals (d) by MR-CpGCluster.

### 3.2 Data annotation

Data annotation is described by the right side of Figure 1. Firstly, the position-defined CGIs are classified into different densities by Supplementary Eq. S2. And then, we classify each CpG methylation site of CGIs into different gene functional regions by Supplementary Table S2. Lastly, we classify the CpG sites into three methylation levels by Eq.5.

**FIGURE 3:**
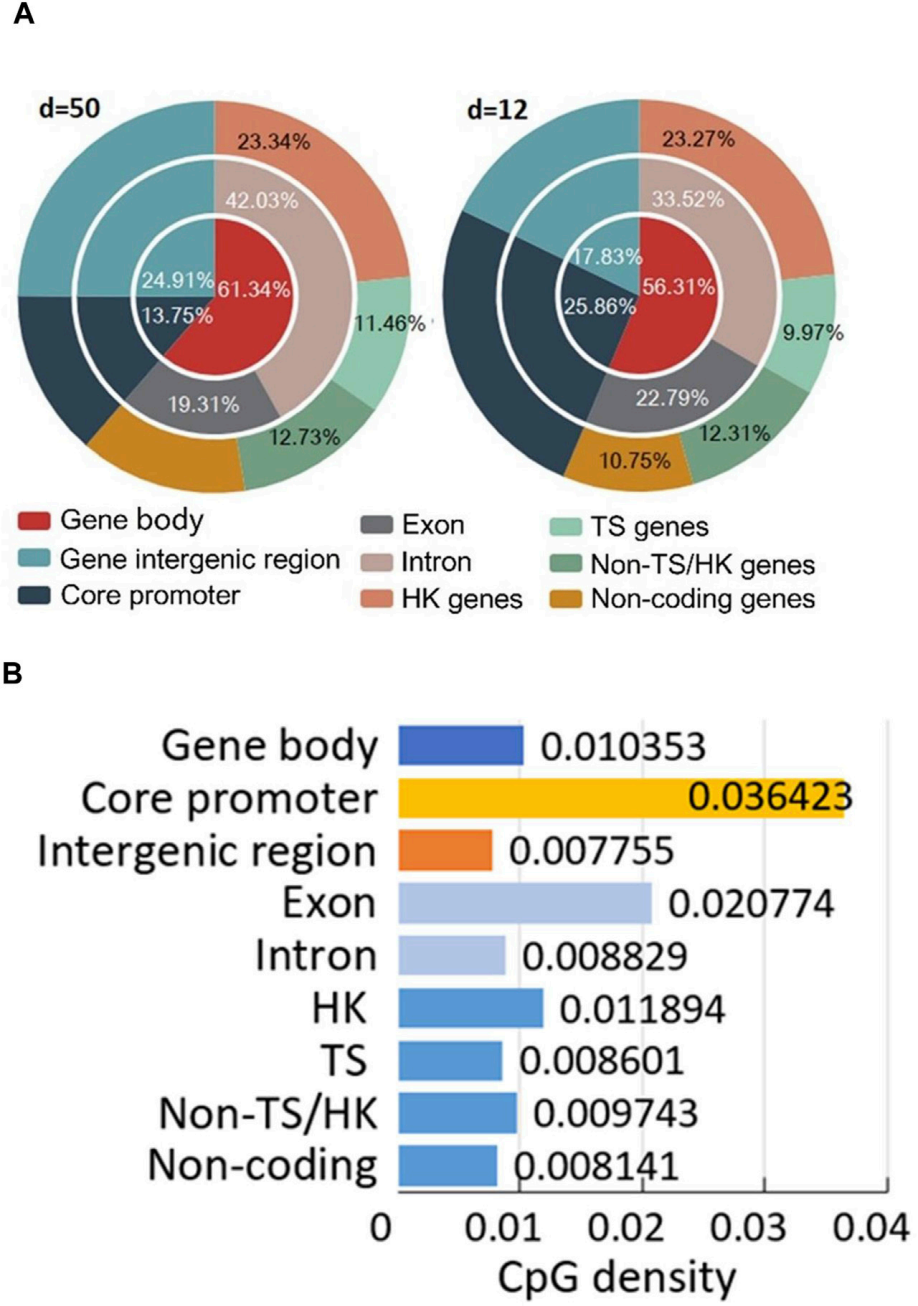
Position-defined CGI annotation results. **(A)** Distribution of all CpG sites in different structural and functional categories of genome sequences. **(B)** CpG density (Eq. 4) of different gene and sequence categories.

Data annotation can help us investigate the distribution of all CpG sites in different structural and functional categories of genome sequences (Figure 3; Supplementary Table S3). For example, we not only can compare the distribution of the number of CpG sites in each region of the predicted CGIs under different CpG interval(d) (Figure 3A), but also visualize the density of CpG sites in different functional regions (Figure 3B).

### 3.3 Human position-defined CGI methylation analysis

The position-defined CGI methylation analysis is described by the bottom side of Figure 1 with three functions.

**FIGURE 4:**
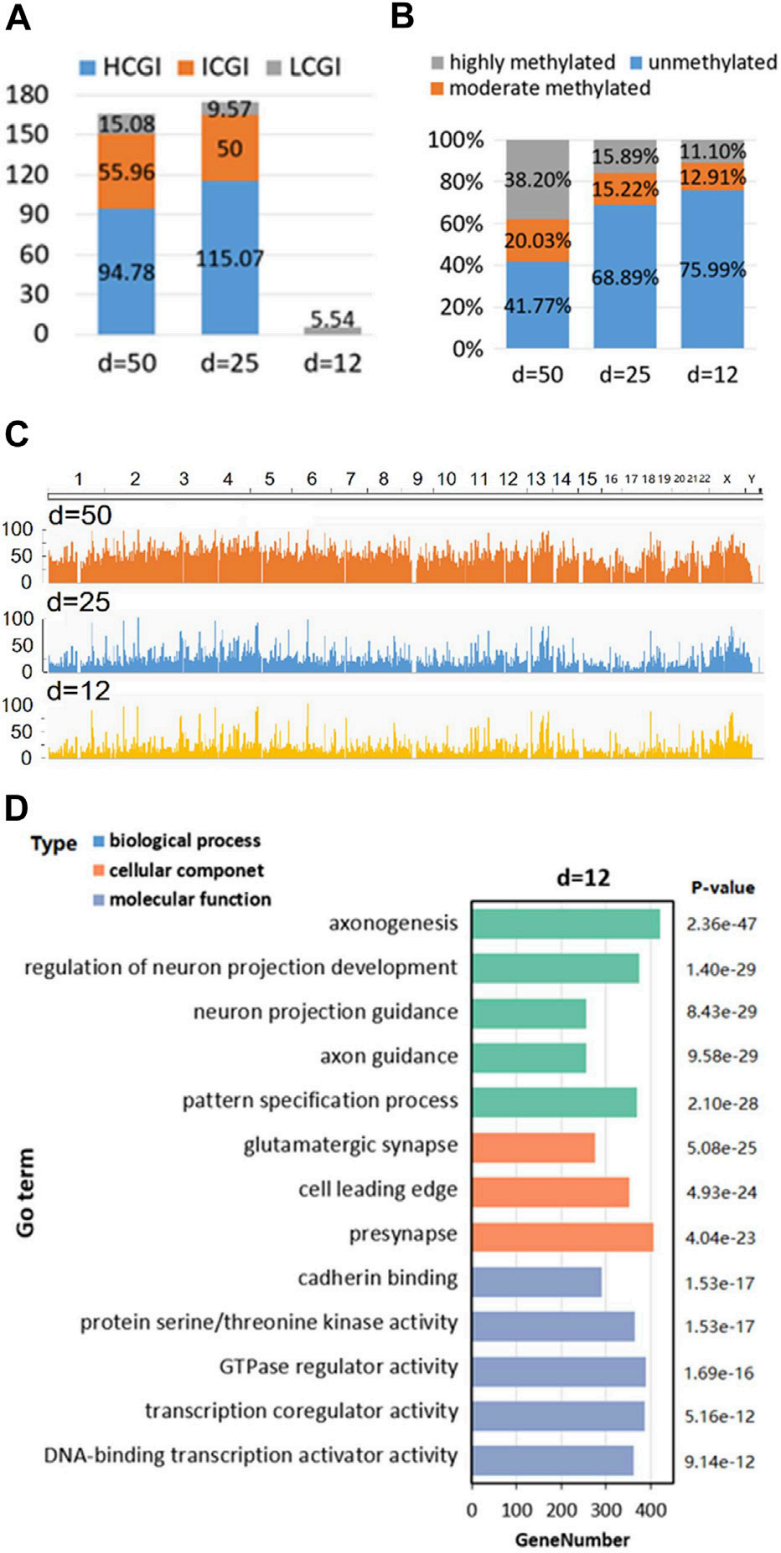
Human position-defined CGI methylation analysis. **(A)** The classification of position-defined CGI under CGI density. **(B)** CpG density of different gene and sequence categories. **(C)** Comparison of the methylation ratio of position-defined CGIs. The horizontal and vertical axes represent genomic chromosome position and the methylation rate of the CpG site at corresponding position, respectively. **(D)** GO enrichment analysis.

First is “CGI density analysis” (Figure 4A), which is used to analyze the classification of position-defined CGI under various CGI density (Weber et al., 2007; Zhu et al., 2008) and CpG interval (d).

Second is “Methylation level analysis,” which not only can analyze the specificity of methylation level for CpG sites under different annotation categories and CpG interval (d) (Figure 4B), but also allows the visualization and comparative analysis of methylation level of position-defined CGIs at the genome-wide perspective (Figure 4C).

The third is “GO enrichment analysis,” which employs clusterProfiler (Yu et al., 2012) to make GO enrichment analysis (Liu et al., 2020) for the CGI + genes (Coding genes that at least one of its TSSs is located in the CGI) (Weber et al., 2007; Zhang et al., 2021a) of position-defined CGIs. Here, Figure 4D shows GO enrichment analysis for the CGI + genes under CpG interval d = 12bp.

### 3.4 Algorithm performance comparison

**FIGURE 5:**
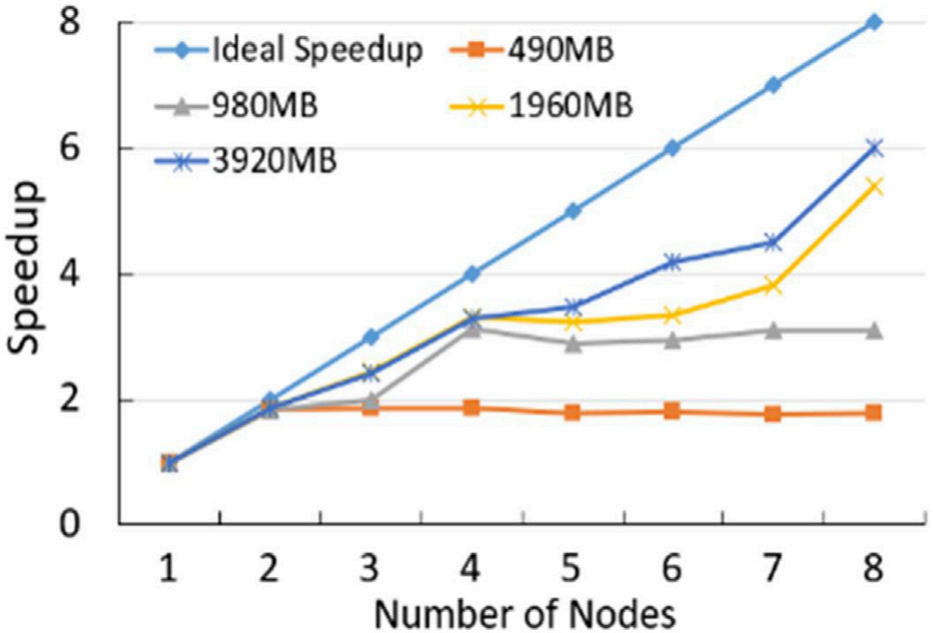
The speedup ratio of MR-CpGCluster.

Firstly, As shown in Figure 5; Supplementary Figure S2, we compare the computing speed for Human position-defined CGI prediction method with MR-CpGCluster and this method without MR-CpGCluster with three commonly used standards: Speedup, Scaleup and Sizeup (Schatz, 2009). Figure 5 shows that the Speedup is positively related to the number of nodes and the size of dataset. For example, when using 8 nodes for a 3920 MB dataset, the ratio between the actual and ideal Speedup is 6.00/8 = 75%, while with 6 nodes for a 980 MB dataset, this ratio is 2.88/6 = 49.17%.

Next, we compare the computing efficiency for Human position-defined CGI prediction method with commonly used density-defined CGIs prediction method (Weber et al., 2007; Zhang et al., 2021a) and another two classical distance-based CGI prediction methods such as WordCluster (Hackenberg et al., 2011) and CpGProD (Ponger and Mouchiroud, 2002) by CGI length, GC content, and O/E ratio (Eq. 3), which are three broadly used standards (Wang and Leung, 2004; Hackenberg et al., 2010).

Table 1; Supplementary Figure S5 not only demonstrate that the average length of CGIs of Human position-defined CGI prediction method (23.7 ± 11.5bp) is statistically shorter, but also the average GC content (89.3% ± 7.5%) and O/E value (1.54 ± 0.27) of Human position-defined CGI prediction method are statistically greater than other prediction methods by statistical test (Zhang et al., 2021b; Zhang et al., 2021d; Gao et al., 2021; Liu et al., 2021; Lai et al., 2022; Song et al., 2022).

**TABLE 1:** CGI prediction methods comparison.

CGI prediction methods	CGI number	Average length ± standard deviation	Average GC ± standard deviation	Average O/E ± standard deviation	Average CpG Density ± standard deviation
Human position-defined	89,063	23.7 ± 11.5	89.3% ± 7.5%	1.54 ± 0.27	0.294 ± 0.066
CGI prediction method					
CpGCluster	198,445	274.7 ± 249.8	63.8% ± 7.6%	0.86 ± 0.27	0.087 ± 0.04
WordCluster	198,703	273.2 ± 246.4	63.8% ± 7.5%	0.86 ± 0.27	0.087 ± 0.04
CpGProD	76,793	1,043.8 ± 761.7	54.6% ± 6.1	0.64 ± 0.1	0.047 ± 0.016
Density-defined CGIs	30,477	774.7 ± 826.9	66.5% ± 4.7%	0.86 ± 0.14	0.094 ± 0.018

Note: Here, we employ default setup for CpG interval, d = 12bp (Zhang et al., 2018; Zhang et al., 2021a).

### 3.5 Web service construction

**FIGURE 6:**
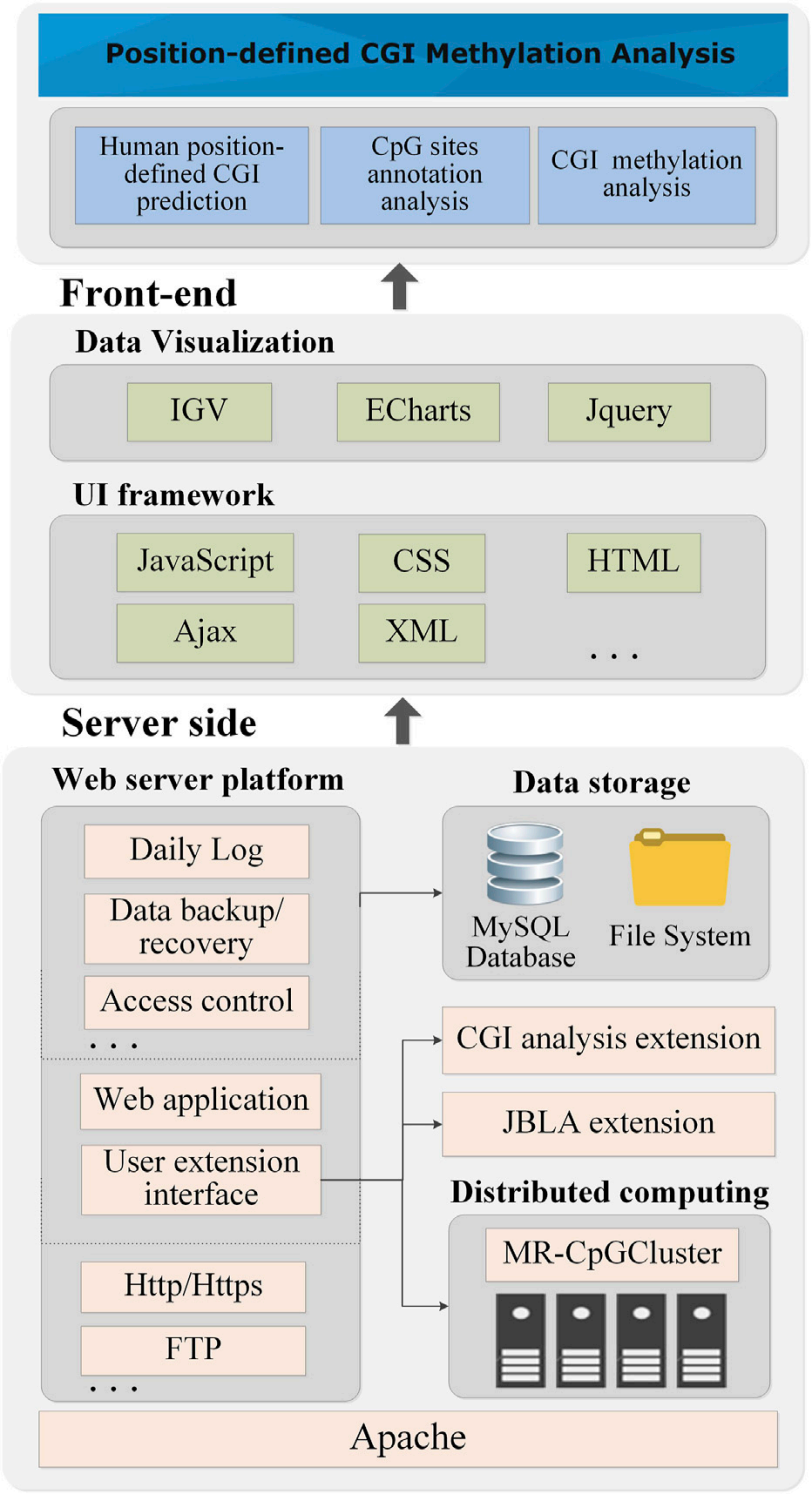
The technical architecture of PCGIMA.

Figure 6 shows the technical architecture of PCGIMA (http://www.combio-lezhang.online/pcgima/home.html), which consists of three modules: “Human position-defined CGI prediction,” “CpG sites annotation analysis,” and “CGI methylation analysis.”

PCGIMA employs MR-CpGCluster to predict the position-defined CGI for multiple consecutive (d) values. To compare and analyze the CpG methylation levels in different genome regions, we integrate the JavaScript version of IGV (Integrative Genomics Viewer) (Thorvaldsdottir et al., 2013) into our Web service. PCGIMA also imports the genome annotation information and analysis results into the MySQL database (Xia et al., 2010) and use eCharts (Bond and Goguen, 2002) to visualize CGI-related analysis results.

“Human position-defined CGI prediction” module provides two functions (Figure 2). One is “Position-defined CGI prediction,” which can online predict position-defined CGI for the human genome or a particular chromosome with multiple consecutive (d) values. The other is “Position-defined CGI features analysis,” which can describe the connection between the proportion distribution of CGI and CGI features.
“CpG sites annotation analysis” module consists of two functions. First is “Human CpG sites Distribution analysis,” which can analyze the distribution of CpG methylation sites in different structural and functional categories of genomic sequences (Figure 3). Second is “Human CpG sites permutation analysis” module (Supplementary Method S4), which can analyze the CpG permutation patterns (Zhang et al., 2018) of density- and position-defined CGIs.
“CGI methylation analysis” module also provides two functions. One is “Position-defined CGI methylation analysis,” which can analyze the specificity of methylation level for CpG sites under different annotation categories (Figure 4B). The other is “GO enrichment analysis,” which can make GO enrichment analysis for the CGI + genes of position-defined CGIs (Figure 4D). Meanwhile, PCGIMA also provides related source code and data download services. The function descriptions are detailed in Supplementary Method S4.

## 4 Discussion and conclusion

This study not only develops Human position-defined CGI prediction method to locate short and position-sensitive CGIs (CpG islets) using high performance computing to construct MR-CpGCluster algorithm (Figure 1), but also a novel human genome annotation and analysis method to investigate the connections among CGI, gene expression and methylation. Finally, we integrate them into PCGIMA for online computing and visualization.

For Human position-defined CGI prediction method, it not only can efficiently locate CpG islets (Figure 2A; Table 1), but also it can parallel predict position-defined CGIs with multiple consecutive (d) values and investigate the genetic characteristics of position-defined CGIs under different CpG interval parameters (Figures 2B–D; Supplementary Datas S1–S3).

For annotation method, it can investigate the connections between position-defined CGI methylation and gene expression specificity from a genome-wide perspective by considering functional regions (core promoters and gene bodies) and the distribution of methylation sites of genes for different expression breadth (Figure 3). Our annotation method (Figure 3A) reveals that the distribution proportion of methylation sites in TS genes for short positional-defined CGIs (d = 12) is 9.97%, which is less than that for long positional-defined CGIs (d = 50, 11.46%).

For Human position-defined CGI methylation analysis, not only CGI density analysis (Figure 4A) finds an interesting phenomena that short position-defined CGIs (CpG islets) are closer to LCGI by classifying the position-defined CGI under various CGI density (Weber et al., 2007; Zhu et al., 2008) and CpG interval (d), but also methylation levels analysis demonstrates that the average methylation levels are obviously low for CpG islets from overall scale and genome-wide perspective, respectively (Figures 4B, C) as well as Go enrichment analysis implies that the position-defined CGI-related genes could be associated with unique gene regulatory functions (Figure 4D; Supplementary Figure S4).

For Algorithm performance comparison, Figure 5 turns out that MR-CpGCluster method is faster than classical CpGCluster for the big dataset, which implies Human position-defined CGI prediction method can parallel process the big CGI data.

Moreover, previous studies indicate that CGIs with length less than 200 bp may be functional CpG islets (Hackenberg et al., 2006) and high GC content and O/E values represent enrichment of methylation sites (Gardinergarden and Frommer, 1987; Takai and Jones, 2002). Since Table 1 demonstrates that the average CGI length of the Human position-defined CGI prediction method is much less than 200bp (column 3 of Table 1), and the average GC content and O/E value are statistically greater than other prediction methods (column 4 and 5 of Table 1), we can conclude that Human position-defined CGI prediction method can locate more potential short CGIs with special functions than previous CGI prediction methods (Takai and Jones, 2002; Takahashi et al., 2017).

Lastly, Figure 6 shows that since we utilize the MR-CpGCluster to speed up CGI prediction and incorporate extensive visualization methods to increase user usability, PCGIMA provides an easy-to-use analysis and visualization platform for human CGI prediction and methylation. It should be noted that since the human genome annotation and analysis results have been computed and imported into the database in advance, it is fast (about 2–3 min) for PCGIMA to show the analysis results except the “Human position-defined CGI prediction.”

Although our study already made great progress in CGI prediction, annotation, analysis, and visualization, it still needs further improving. Firstly, we should make detail annotations for human position-defined CGIs in terms of functional and structural features. Secondly, we should interrogate the lineage-based and function-based subsets for CGIs and their regulatory implications (Blackledge et al., 2013). Finally, we should employ advanced high performance computing technology (Jiang et al., 2015; Zhang et al., 2021c; Xiao et al., 2021) to improve PCGIMA in the distant future.

## Data Availability

The original contributions presented in the study are included in the article/Supplementary Material, further inquiries can be directed to the corresponding author.
