# Supplementary material for: PCGIMA: developing the web server for human position-defined CpG islands methylation analysis
Source: Front Genet. 2024 Mar 13;15:1367731. doi: 10.3389/fgene.2024.1367731 (PMC10965558; doi:10.3389/fgene.2024.1367731)
Supplement: Supplementary file 1 [file Table1.DOCX]

Supplementary Material

Supplemental Material includes:

# Supplementary methods

**Method S1.** MR-CpGCluster.

**Method S2.** Methylation ratio computation of CpG sites.

**Method S3.** CGI density and sequence region annotation.

**Method S4.** The performance of PCGIMA.

# Supplementary figures

**Figure S1.** Flow chart of MR-CpGCluster.

**Figure S2.** Parallel performance of MR-CpGCluster.

**Figure S3.** The average methylation ratio of position-defined CGIs.

**Figure S4.** Differential gene distributions of three position-defined CGIs.

**Figure S5.** CGI prediction methods Comparison.

# Supplementary tables

**Table S1.** Information of 29 tissues used to compute the CpG methylation rate.

**Table S2.** The annotation standard of sequence regions.

**Table S3.** The CpG site distribution over gene structures and CGI definitions.

# Supplementary data

**Data S1.** The 50-bp position-defined CGIs.

**Data S2.** The 25-bp position-defined CGIs.

**Data S3.** The 12-bp position-defined CGIs.

# Supplementary methods

**Method S1.** MR-CpGCluster

As shown in Fig.S1, the main steps of the MR-CpGCluster framework are as follows:

**1. Extraction and preprocessing of genomic data.** Sequence data were extracted from the genetic genome database (Step 1.1), classified by chromosome (Step 1.2), and preprocessed. The sequence data is then stored in HDFS. Hadoop divides the data into blocks based on the block size (here we set it as 256MB) and stores the data in a distributed mode (step 1.3).

**2. Use MapReduce to compute distributed CpGcluster results.** Like the normal MapReduce architecture, MapReduce manages the mapping of file blocks and then starts the Master node to manage Mapper and Reducer tasks. First, MapReduce sends Map tasks to different Mapper nodes and splits them according to chromosome numbers (Step 2.1). Second, the Mappers node receives split data from the Master, starts the Hadoop Streaming process and passes the data to the Streaming as standard input (Step 2.1.1); Streaming runs the CpGcluster algorithm in any language and outputs the local results as key-value pairs using the standard method; To Mapper node (Step 2.1.2); The master node triggers Reducers to process the chromosome clustering results of single nodes generated by Mapper nodes (Step 2.2), and merge the clustering results of complete human genome (Step 2.3).

**3. CGI statistics and analysis.** All data of human genome were obtained from MapReduce (Step 3.1), and CpGcluster results were analyzed (Step 3.2) for subsequent further CGIs analysis (Step 4).

**Fig. S1.** Flow chart of MR-CpGCluster.

We use the real human genome dataset GRCH38 to conduct the parallel performance experiment and analysis of MR-CPGCluster. In the experiment, the parameter d in CPGCluster is selected as 12. Then, three commonly used standards are used to evaluate the performance of MR-CPGCluster: Speedup, Scaleup, and Sizeup (Park, et al., 2016).

The source code of MR-CpGCluster can be download from: [www.combio-lezhang.online:8080/supplementary_data/MR_CpGCluster_code.zip](http://www.combio-lezhang.online:8080/supplementary_data/MR_CpGCluster_code.zip)

**Fig. S2.** Parallel performance of MR-CpGCluster. (A) Speedup ratio; (B) Sizeup ratio; (C) Scaleup ratio.

Fig. S2A shows that the Speedup is positively correlated with the number of nodes when the amount of data is large. For example, when using 8 nodes to compute 490MB data set, the ratio of actual to ideal speedup is 4.18/8 = 52.25%, while when using 8 nodes to compute 1960MB data set, the ratio is 6/8 = 75%. Fig. S2B shows for the same data size, Sizeup is negatively correlated with the number of nodes. For example, when the data is 3920MB, the Sizeup of 2 nodes is 7.59 and the Sizeup of 6 nodes is 3.31. As shown in Fig S2C, Scaleup is negatively correlated with the number of nodes and the amount of data. Taking 5 nodes as an example, when the data increases from 245MB to 1960MB, the scaleup is 0.89. However, the Scaleup decreases to 0.78 when the data increases from 980MB to 7840MB.

**Method S2.** Methylation ratio computation of CpG sites.

To investigate methylation state of CGIs in different sequence regions, we calculated the average methylation ratio at each CpG site of human genome by Eq. 4 and CpG methylation data of 29 human tissues (Table S1).

$\begin{aligned} \mathbf{methylatio}\mathbf{n}_{\mathbf{r}}\mathbf{atio}\left( \boldsymbol{chr,p} \right)\boldsymbol{=}\frac{\sum_{\boldsymbol{t=1}}^{\boldsymbol{29}} \mathbf{methylation}\left( \boldsymbol{chr,p,t} \right)}{\mathbf{29}}\boldsymbol{\#}\left( \boldsymbol{1} \right) \end{aligned}$

Here, chr and p represent the chromosome and position of the CpG site, respectively. t is the index of tissue. Methylation(chr, p, t) represents the methylation ratio of the CpG site at position p of chromosome chr on tissue t.

**Method S3.** CGI density and sequence region annotation

# CGI density annotation

According to the CGI density annotation method(Weber, et al., 2007; Xiao, et al., 2019, DOI:10.1109/TCBB.2019.2935971), we annotate each CGI as HCGI, ICGI and LCGI by Eq. 3 (Xiao, et al., 2019, DOI:10.1109/TCBB.2019.2935971).

$\begin{aligned} \text{CGIDensity}\text{ }\mathbf{=}\left\{ \begin{aligned} \boldsymbol{&}\text{1, HCGI }\boldsymbol{N\geq}\text{500,GCRatio}\boldsymbol{\geq0.6,}\text{O/E}\boldsymbol{\geq0.75} \\ \boldsymbol{&}\text{2, ICGI }\boldsymbol{N\geq}\text{500,GCRatio}\boldsymbol{\geq0.5,}\text{O/E}\boldsymbol{\geq0.48}\text{ } \\ \boldsymbol{&}\text{3, LCGI otherwise } \end{aligned} \right.\boldsymbol{\#}\left( \boldsymbol{2} \right) \end{aligned}$

Here, CGIDensity represents the density classification of a CGI, N is the length of CGI, GCRatio represents GC content ratio of the CGI.

# Sequence region annotation

Based on the data from GENCODE(Wright, et al., 2016), we investigate the distribution of CpG sites in different sequence regions as follows. First, we annotate sequence regions of human genome into gene body, gene intergenic region and core promoter. Furthermore, gene body is classified into intron and exon (Clark, et al., 2016). Secondly, according to the previous studies(Xiao, et al., 2019, DOI:10.1109/TCBB.2019.2935971; Zhang, et al., 2019, DOI:10.1093/bib/bbz134), we classified the gene body into four categories: TS (tissue-specific), HK (house-keeping), non-TS/HK and non-coding genes according to tissue expression specificity. Table S2 lists the annotation standard.

**Method S4.** The performance of PCGIMA.

See “Supplementary file S1_The performance of PCGIMA.docx” for detail.

# Supplementary figures

**Fig. S1.** Flow chart of MR-CpGCluster.

A. B.


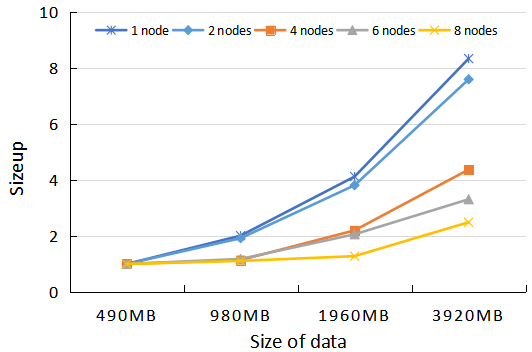

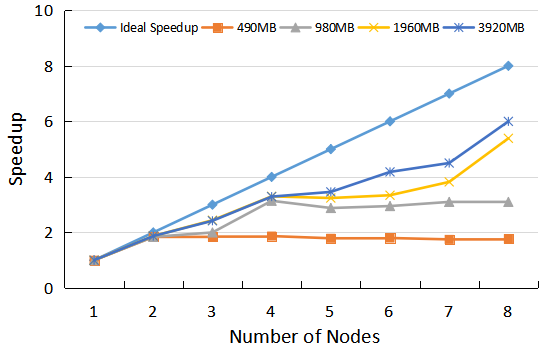


C.


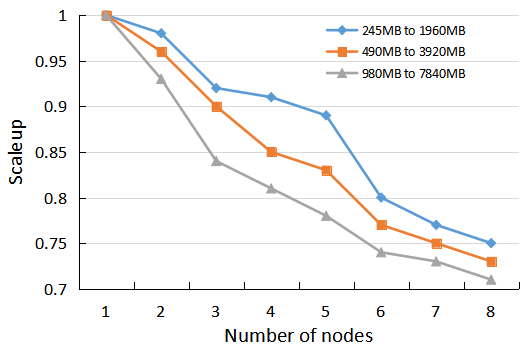


**Fig. S2.** Parallel performance of MR-CpGCluster. (A) Speedup ratio; (B) Sizeup ratio; (C) Scaleup ratio.

1. B.


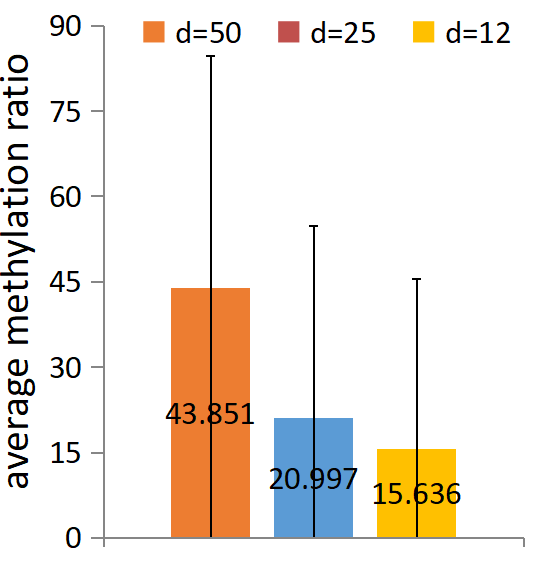

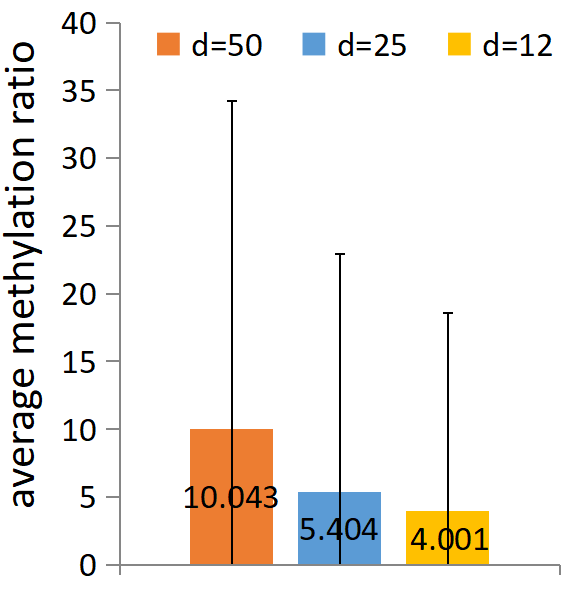


**Fig. S3.** The average methylation ratio of position-defined CGIs. (A) The average methylation ratio of position-defined CGIs for all position-defined CGIs. (B) The average methylation ratio of position-defined CGIs for position-defined CGIs intersecting core promoters.


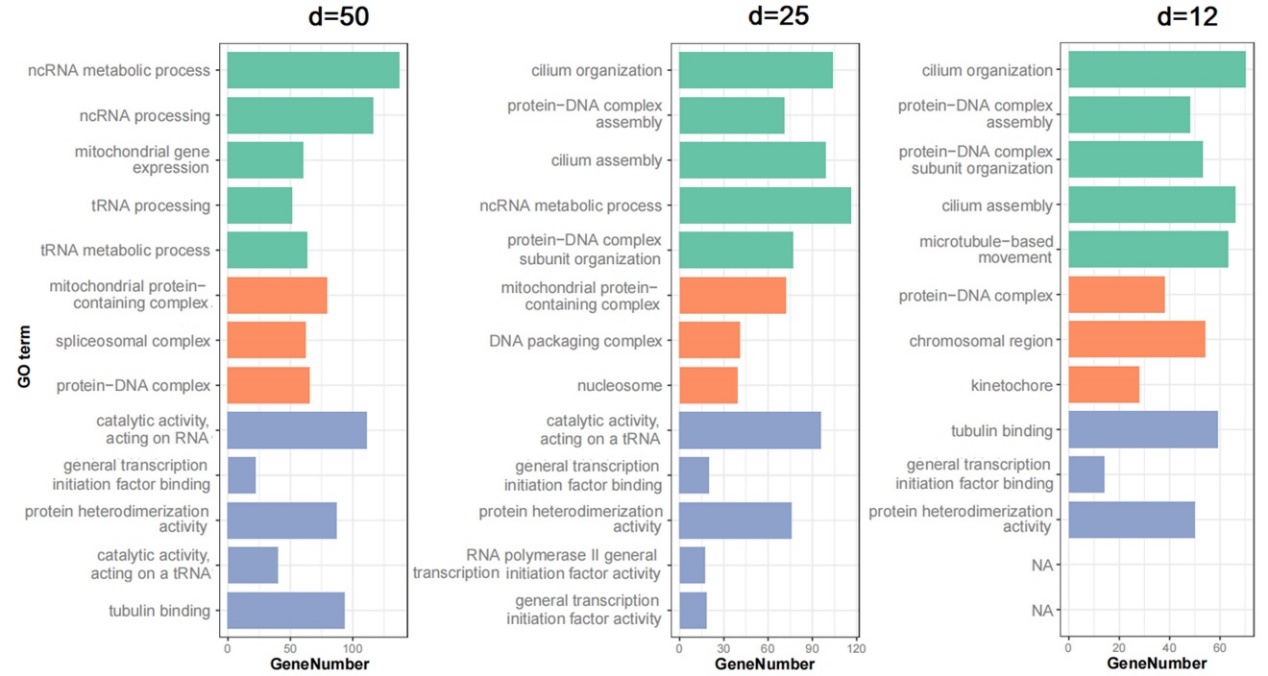


**Fig.S4.** Differential gene distributions of three position-defined CGIs. Human genes which overlap with position-defined CGIs are categorized based on GO (Yu, et al., 2012) terms in molecular function, cellular component, and biological process, and only the top few enriched categories are used for the comparison. Note that most of the terms are shared by the three CGI groups but some appear unique to only one of them, where spliceosomal complex, DNA packaging complex, and kinetochore of cellular components are found only in 50-bp, 25-bp, and 12-bp subgroups, respectively.

A.


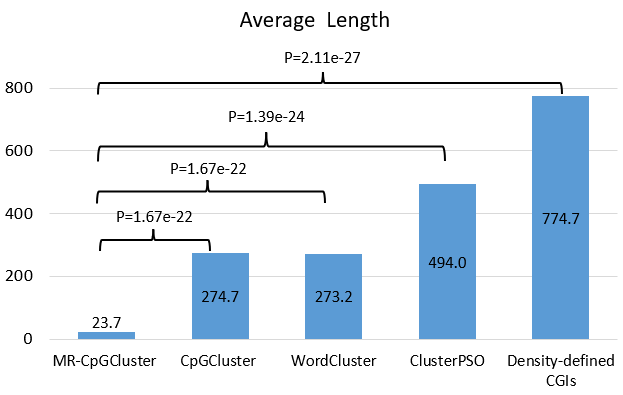


B.


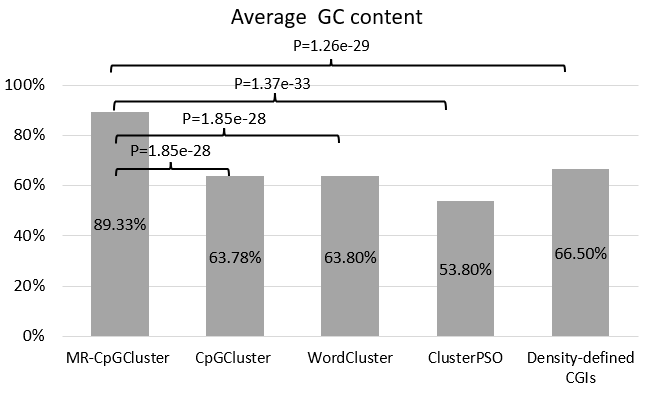


C.


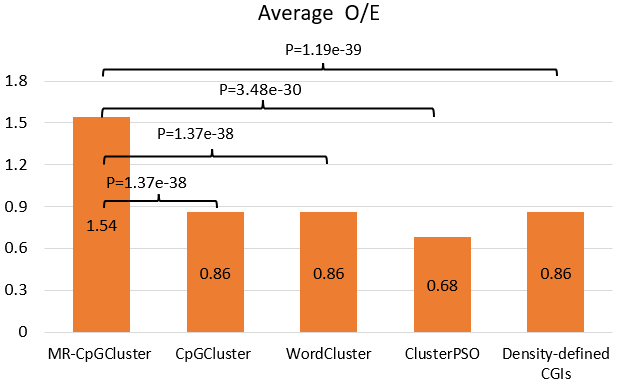


**Fig. S5.** CGI prediction methods comparison. Comparison for (A) Average length, (B) Average GC content, (C) Average O/E. In the figure, p represents the p-value of T-Test.

# Supplementary tables

# TABLE S1

Information of 29 tissues used to compute the CpG methylation rate.

| Tissue name | File accession ID | Tissue name | File accession ID |
| --- | --- | --- | --- |
| adipose tissue | ENCFF318AMC | **lung** | ENCFF039JFT |
| adrenal gland | ENCFF913ZNZ | **muscle of leg** | ENCFF588ETU |
| aorta | ENCFF553HJV | **muscle of trunk** | ENCFF645AZF |
| body of pancreas | ENCFF748MTS | **ovary** | ENCFF303ZGP |
| esophagus | ENCFF625GVK | **pancreas** | ENCFF763RUE |
| heart | ENCFF560SMW | **placenta** | ENCFF437OKM |
| heart left ventricle | ENCFF536RSX | **prostate gland** | ENCFF027KTR |
| heart right ventricle | ENCFF513ITC | **psoas muscle** | ENCFF913UZU |
| large intestine | ENCFF923CZC | **right cardiac atrium** | ENCFF110AZO |
| lower leg skin | ENCFF121VIX | **sigmoid colon** | ENCFF157POM |
| small intestine | ENCFF521DHD | **thymus** | ENCFF392XPZ |
| spinal cord | ENCFF164EAU | **thyroid gland** | ENCFF497IYX |
| spleen | ENCFF550FZT | **tibial nerve** | ENCFF699KTW |
| stomach | ENCFF811QOG | **upper lobe of left lung** | ENCFF733EFJ |
| testis | ENCFF638QVP |  |  |

# TABLE S2.

The annotation standard of sequence regions.

| Definition | Description |
| --- | --- |
| Gene body | The sequence region that annotated as “gene” in GENCODE human genome annotated data. |
| Gene intergenic region | The intergenic region among each gene body region |
| Core promoter | [-100, +100] region around each TSS |
| Exon | The sequence region that annotated as “exon” in gene body. |
| Intron | The intergenic region among each exon in gene body. |
| TS genes | Genes that are expressed in a few tissues to perform unique functions. |
| Non-TS/HK genes | Those coding genes that are neither TS nor HK. |
| HK genes | Genes that are expressed in almost all tissues to maintain basic cellular functions. |
| Non-coding genes | Genes that produce functional RNAs instead of encoding proteins. |

# TABLE S3.

The CpG site distribution over gene structures and CGI definitions

| Sequences | All CpGs | Gene body CpGs | Core promotor CpGs |
| --- | --- | --- | --- |
| Genome (GRCh38) | 27,340,636 | 17,238,783 | 956,267 |
| Position-defined  All CGIs | 4,095,469(14.98%) | 4,095,469(23.76%) | 650,637 (68.04%) |
| Position-defined CGIs d=50 | 4,551,379 (16.65%) | 2,900,488 (16.83%) | 650,211 (67.99%) |
| Position-defined CGIs d=25 | 1,594,162 (5.83%) | 1,026,393 (5.95%) | 382,013 (39.95%) |
| Position-defined CGIs d=12 | 580,431 (2.12%) | 332,629 (1.93%) | 152,746 (15.97%) |
| Density-defined  all CGIs | 2,207,802(8.08%)） | 1,616,177 (9.38%) | 573,986 (60.02%) |
| Density-defined HCGI | 1,582,957 (5.79%) | 1,196,023 (6.94%) | 457,544 (47.85%) |
| Density-defined ICGI | 233,132 (0.85%) | 161,082 (0.93%) | 35,853 (3.75%) |
| Density-defined LCGI | 391,713 (1.43%) | 259,072 (1.5%) | 80,589 (8.43%) |

*Note:* the proportions of each group within the categories are calculated and placed in the parentheses next to the numbers.

**Supplementary data**

**Data S1.** The 50-bp position-defined CGIs results can be download from: [www.combio-lezhang.online/PCGIMA/supplementary_data/S1_d50_all_CGIs.bed](http://www.combio-lezhang.online/PCGIMA/supplementary_data/S1_d50_all_CGIs.bed).

**Data S2.** The 25-bp position-defined CGIs results can be download from: [www.combio-lezhang.online/PCGIMA/supplementary_data/S2_d25_all_CGIs.bed](http://www.combio-lezhang.online/PCGIMA/supplementary_data/S2_d25_all_CGIs.bed).

**Data S3.** The 12-bp position-defined CGIs results can be download from: [www.combio-lezhang.online/PCGIMA/supplementary_data/S3_d12_all_CGIs.bed](http://www.combio-lezhang.online/PCGIMA/supplementary_data/S3_d12_all_CGIs.bed).

References

Clark, K.*, et al.* GenBank. *Nucleic Acids Research* 2016;44(Database issue):D67-D72.

Park, D., Wang, J. and Kee, Y.S. In-Storage Computing for Hadoop MapReduce Framework: Challenges and Possibilities. *IEEE Transactions on Computers* 2016;PP(99):1-1.

Weber, M.*, et al.* Distribution, silencing potential and evolutionary impact of promoter DNA methylation in the human genome. *Nature Genetics* 2007;39(4):457-466.

Wright, J.C.*, et al.* Improving GENCODE reference gene annotation using a high-stringency proteogenomics workflow. *Nature Communications* 2016;7:11778.

Xiao, M.*, et al.* CGIDLA：Developing the Web Server for CpG Island related Density and LAUPs (Lineage-associated Underrepresented Permutations) Study. *IEEE/ACM Transactions on Computational Biology & Bioinformatics* 2019, DOI:10.1109/TCBB.2019.2935971;17(6):2148-2154.

Yu, G.*, et al.* clusterProfiler: an R Package for Comparing Biological Themes Among Gene Clusters. *Omics-a Journal of Integrative Biology* 2012;16(5):284-287.

Zhang, L.*, et al.* CpG-Island-based annotation and analysis of human house-keeping genes. *Briefings in Bioinformatics* 2019, DOI:10.1093/bib/bbz134.
