# Supplementary material for: PCGIMA: developing the web server for human position-defined CpG islands methylation analysis
Source: Front Genet. 2024 Mar 13;15:1367731. doi: 10.3389/fgene.2024.1367731 (PMC10965558; doi:10.3389/fgene.2024.1367731)
Supplement: Supplementary file 2 [file Table2.DOCX]

# Performance of PCGIMA

The homepage of PCGIMA (http://www.combio-lezhang.online/pcgima/HOME.html) is shown in Fig. 1. The corresponding interface will be displayed on the middle when user clicks one of these links in the function navigation bar on the top (Fig. 1).


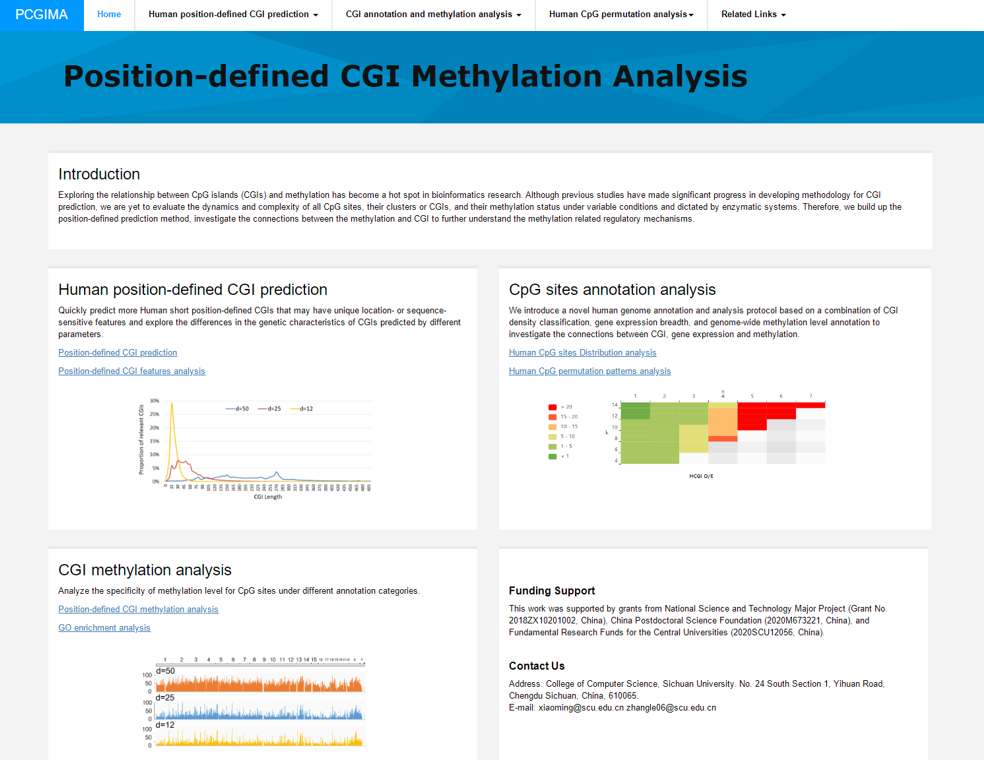


**Fig.1** The homepage of PCGIMA.

"Human position-defined CGI prediction" module provides two functions. One is "Position-defined CGI prediction", which can online predict position-defined CGI for the human genome or a particular chromosome with multiple consecutive (d) values. The other is "Position-defined CGI features analysis”, which can describe the connection between the proportion distribution of CGI and CGI features.

"CpG sites annotation analysis" module consists of two functions. First is “Human CpG sites Distribution analysis”, which can analyze the distribution of CpG methylation sites in different structural and functional categories of genomic sequences. Second is "Human CpG sites permutation analysis" module, which can analyze the CpG permutation patterns of density- and position- defined CGIs.

"CGI methylation analysis " module also provides two functions. One is “Position-defined CGI methylation analysis”, which can analyze the specificity of methylation level for CpG sites under different annotation categories. The other is “GO enrichment analysis”, which can make GO enrichment analysis for the CGI+ genes of position-defined CGIs. The next sections will introduce these links in detail.

# Human position-defined CGI prediction

# 1.1 Position-defined CGI prediction

After clicking " CpG island Cluster Counting" link, the method of MR-CpGCluster is briefly introduced at the button of the page (Fig. 2A). The code of MR-CpGCluster is available to download when user clicks the “here” link (Fig. 2A). User can use the parameter selection interface (Fig. 2B) to select the genome annotation version, the maximum distance parameter range and the chromosome for CGI prediction. Considering the calculation time and the simplicity of analysis result charts, we set 5 as the maximum range of different d for each count (Fig. 2B). User can get the analysis results by clicking the “submit” button.

MR-CpGCluster count may take several minutes to run. After that, the analysis results (Fig 2C,D) will display at the bottom of the page, user can download specific results data by clicking the "Download data" button.


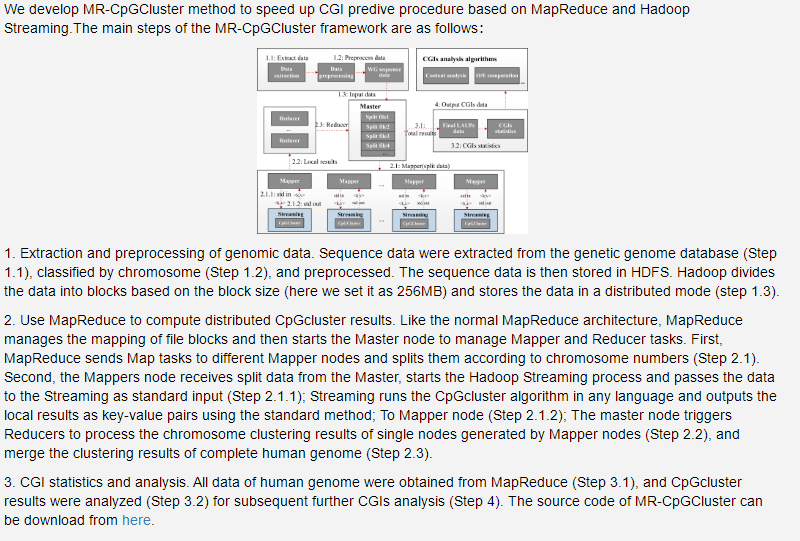


(A)


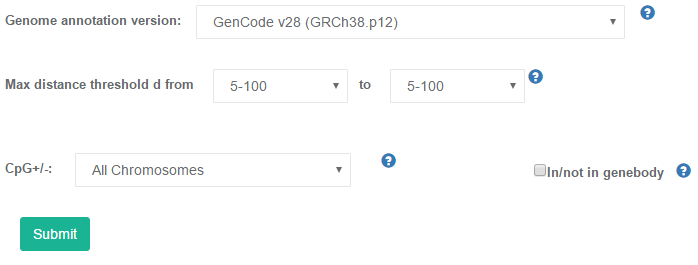


(B)


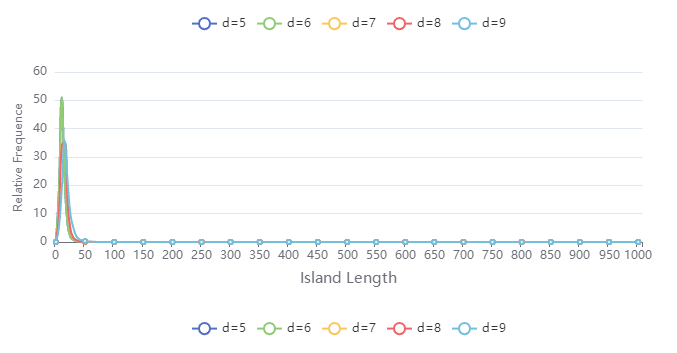


(C)


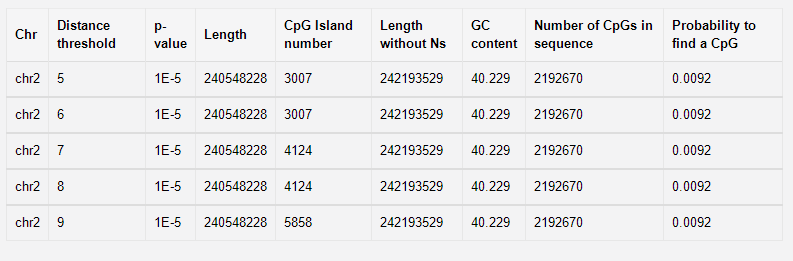


(D)

**Fig.2** Position-defined CGI prediction. (A) Introduction of MR-CpGCluster; (B) The selection interface; (C) Distributions of the island length for the result; (D) The statistical table of the results.

# 1.2 Human position-defined CGI analysis

After clicking "Human position-defined CGI analysis " link, user can use the parameter selection interface (Fig. 3A) to select the genome annotation version, the maximum distance parameter, and the chromosome for MR-CpGCluster analysis. User can also click the radio button to choose whether to distinguish the CGI of in/ not in gene body in the result (Fig. 3A). User can click the submit button to get the analysis results (Fig. 3A).

The analysis results consist of four charts, which show the distribution of island length, CpG O/E ratio, GC content and CpG density of the CGIs which predicted by MR-CpGCluster respectively. Fig. 3F shows some statistical results of the CpGCluster analysis. User can also click the "Download data" button to download specific CpGCluster Analysis results data (Fig. 3F).

For example, after selecting "Gencode v28", "d = 50, d = 25, d = 12", "All Chromosomes" in figure B. Figures C-F show that when d=50, the island length is the longer than d = 25 and d = 12 (Fig.3B), and when d=12, the O/E ratio (Fig. 3C), GC content (Fig. 3D) and CpG density (Fig. 3E) of the CGIs are larger than when d=25 and d=50. Fig. G shows that the less CGI is predicted when d is smaller.


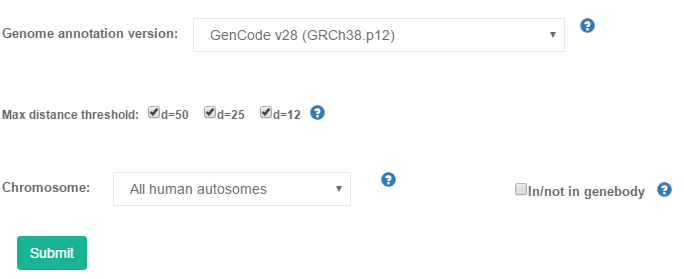


(A)


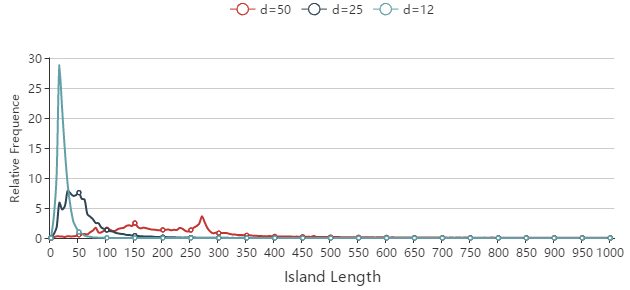


(B)


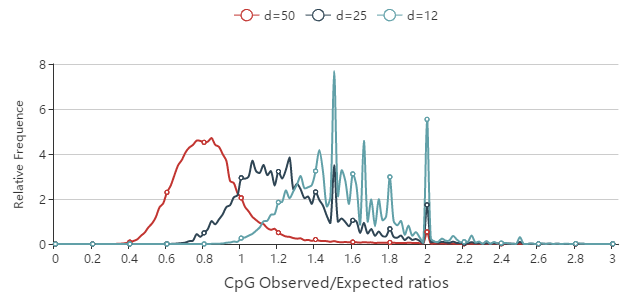


(C)


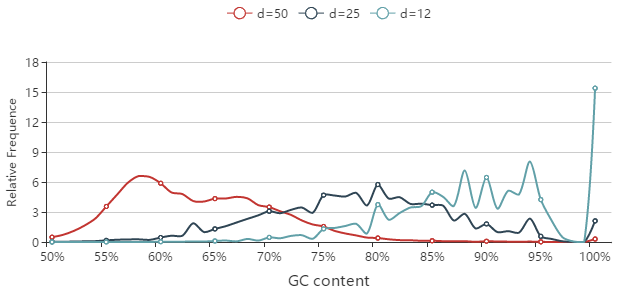


(D)


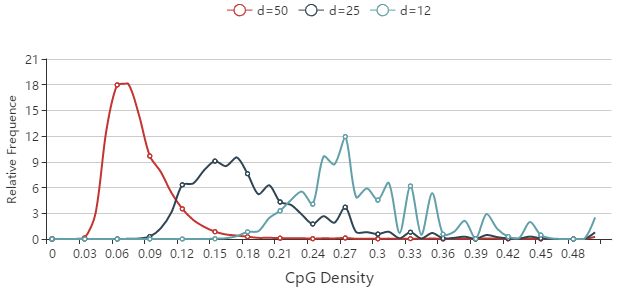


(E)


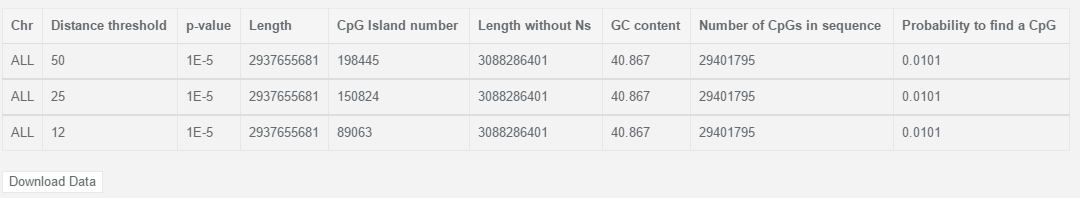


(F)

**Fig.3** Human position-defined CGI analysis. (A) The selection interface; (B) Distributions of the island length; (C) Distributions of the CpG O/E ratio f; (D) Distributions of the GC content; (E) Distributions of the CpG density; (F) The statistical table of cluster results.

# CpG sites annotation analysis

# 2.1 Human CpG sites Distribution analysis

User can employ the interface (Fig. 4A) to choose the genome annotation version and the chromosome after clicking the “Human CpG sites Distribution analysis” link. User can have the CpG sites mapping results by clicking “Submit” button.

The CpG sites mapping results consist of five charts. First, Fig. 4B shows the proportion of CpG sits in different genetic region for the current human genome annotation data. Second, Fig. 4C shows the proportion of CpG sites in different tissue expression specific genes. Third, Fig. 4D shows the density of CpG sites in different gene regions. Fourth, Fig. 4E compares the proportion of CpG sits in different density CGIs. Lastly, Fig. 4F shows average CpG sits number in different density CGIs.

For example, after choosing "Gencode v28", and "All Chromosomes" (Fig. A), Fig. B shows that most CpG sites are locate in the gene body (62.64%). Fig. C shows that most of the CpG sites are locate in the body of coding genes, among which the proportion of HK gene is the highest (20.79%). Fig. D shows that when d=25, the average CpG number in HCGIs is the largest.


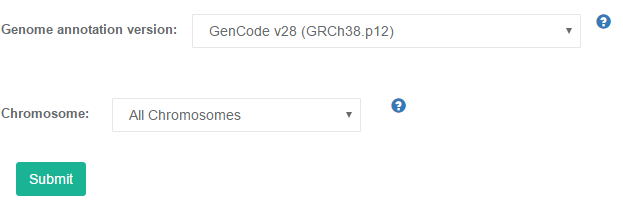


（A）


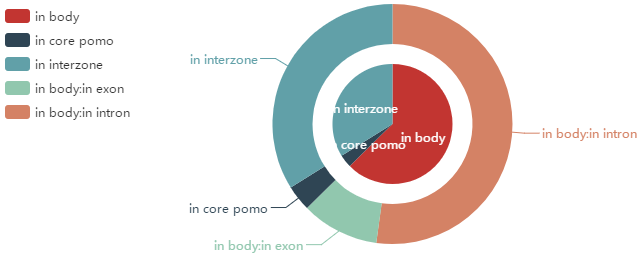


（B）


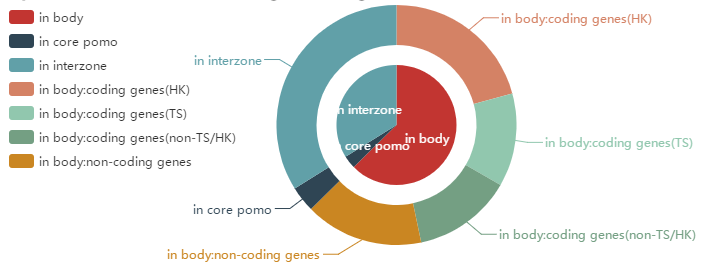


（C）


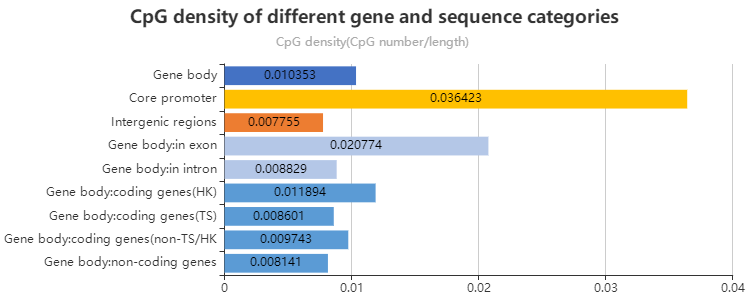


(D)


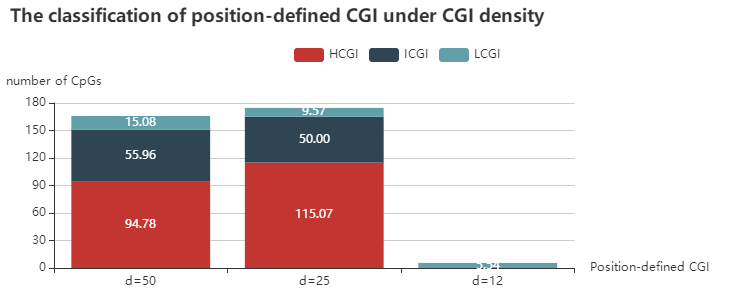


(E)

**Fig.4** Human CpG sites mapping results. (A) The selection interface; (B) The proportion of CpG sits in different genetic region; (C) The proportion of CpG sites in different tissue expression specific genes; (D) Density of CpG sites in different gene regions; (E) Average CpG sits number in different density CGIs.

# 2.2 Human CpG sites permutation analysis

After clicking " Human CpG sites permutation analysis " link, Firstly, the analysis method of CpG-containing sequences permutation patterns analysis was briefly introduced at the button of the page (Fig. 5A). Users can employ the gene annotation category interface (Fig. 5B) to choose the genome annotation version and CGI density. Also, users can click the radio button (Fig. 5B) to show the results of O/E Ratio or O/E Compare. Finally, users can get the analysis results by clicking the "Submit" button (Fig. 5B).


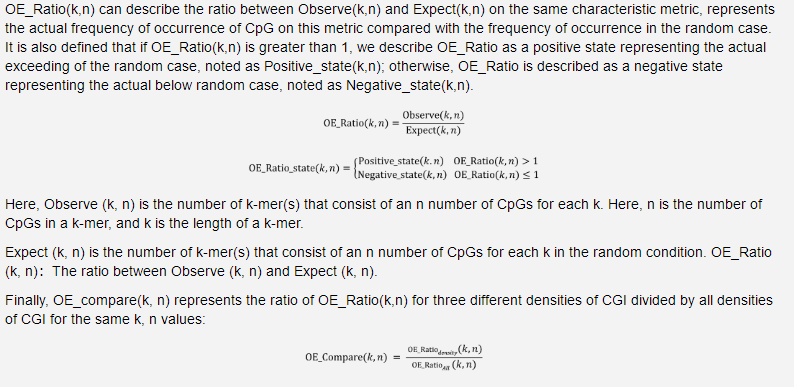


(A)


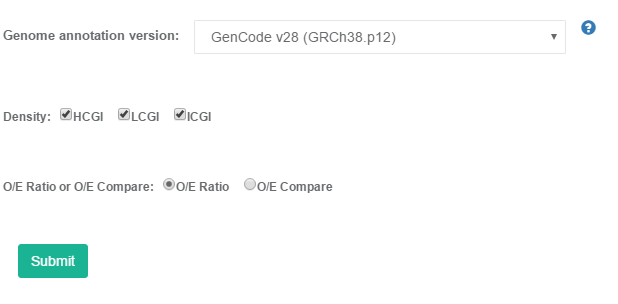


（B）


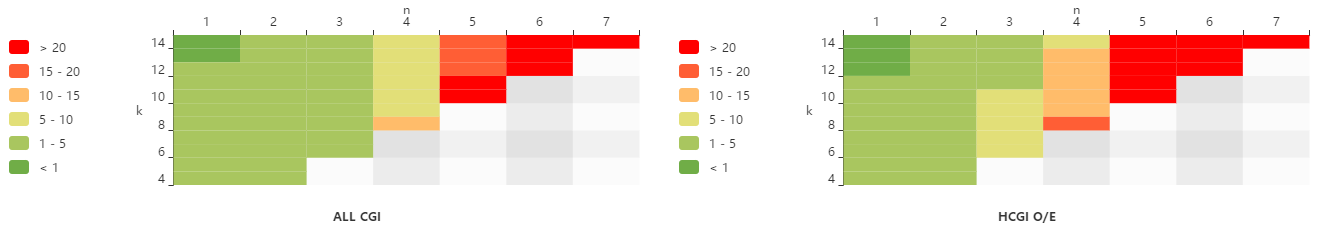


（C）


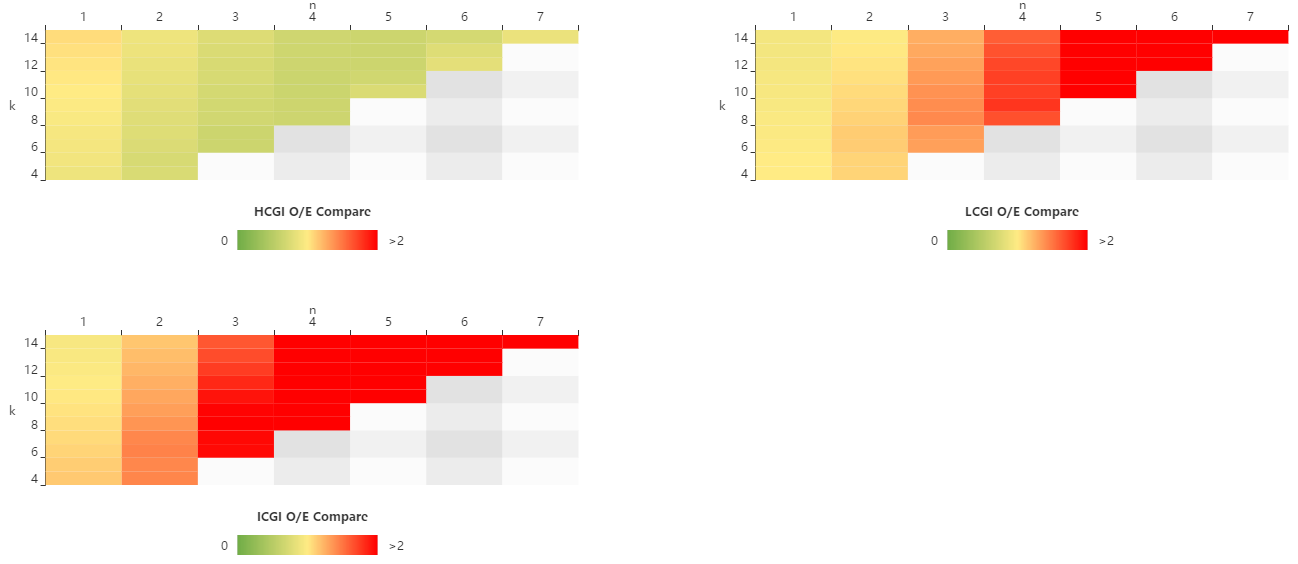


（D）

Fig. 5 CpG permutation patterns analysis. (A) Introduction of CpG-containing sequences permutation patterns analysis; (B) The selection interface for gene annotation category; (C) O/E Ratio for different CGI density groups. (C) OECopmare patterns for different CGI density groups.

The analysis results consist of several heat maps. If user select the radio button of “O/E Ratio”, it will show the O/E Ratio heatmap of all CGIs and HCGIs/ICGIs/LCGIs (Fig. C). If user select O/E Compare, the results are O/E Compare heatmaps of HCGIs/ICGIs/LCGIs (Fig. D).

For example, after selecting "Gencode v28", "HCGI, LCGI, ICGI" and "O/E Compare" in Fig. B, Fig. D shows that OECopmare(k,n) value of HCGIs is close to 1, while OECopmare(k,n) value of ICGIs and LCGIs is positively correlated with n. This indicates that the OERatio mode of HCGIs is very similar to that of all CGIs while the OERatio mode of ICGIs and LCGIs is very different from all CGIs.

# CGI methylation analysis

# 3.1 Position-defined CGI methylation analysis

After clicking "Methylation analysis of Human CGIs " link, user can use the selection interface (Fig. 6A) to select the genome annotation version, the maximum distance parameter, genetic regions intersecting with CGIs and the chromosome for CGIs methylation analysis. User can obtain the analytical results by clicking the “Submit” button (Fig. 6A).

The analytical results consist of three charts. First, Fig. 6B shows the density of CGIs intersecting with different gene regions. Second, Fig. 6C describes the distribution density of CGIs intersecting with gene body. The third one shows the mean methylation level of all CpG sites in CGIs (Fig. 6D). And user can download the average methylation level data by click the links in Fig. 6E.

For example, after selecting "Gencode v28", "d=50, d=25, d=12", “Gene body, Intron, Exon, Core promoter” and "All Chromosomes" in Fig. A, Fig. B shows when d=25, the intersection of CGIs and core promoter is more prominent. Fig. C shows each body region has the highest distribution density of d=50, followed by d=25 and d=12. And Fig. D shows the average methylation level of CGIs predicted by CpGCluster is low when d is small.


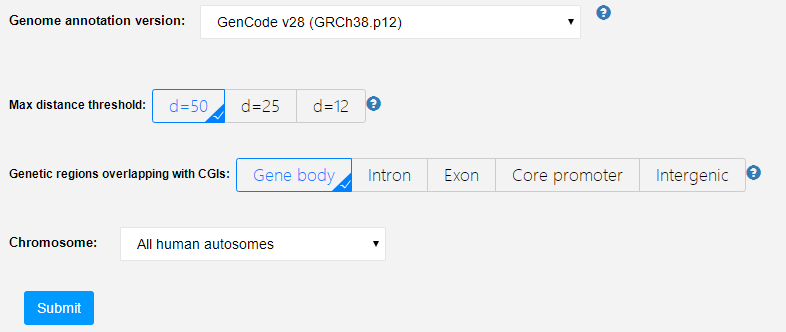


(A)


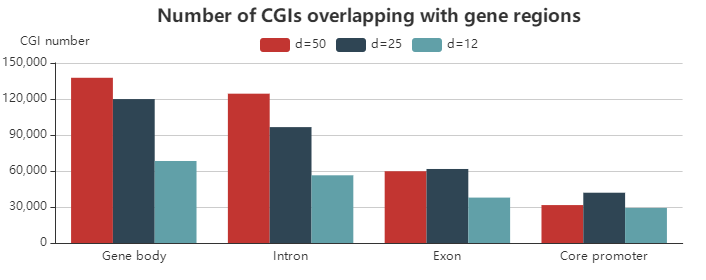


(B)


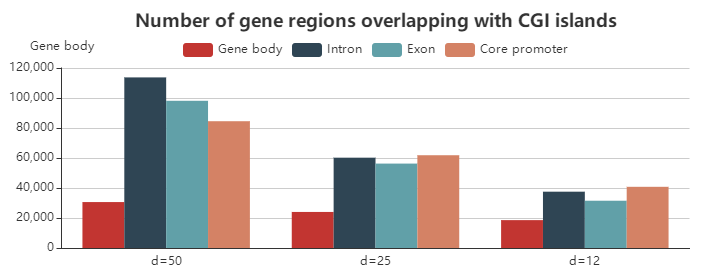


(C)


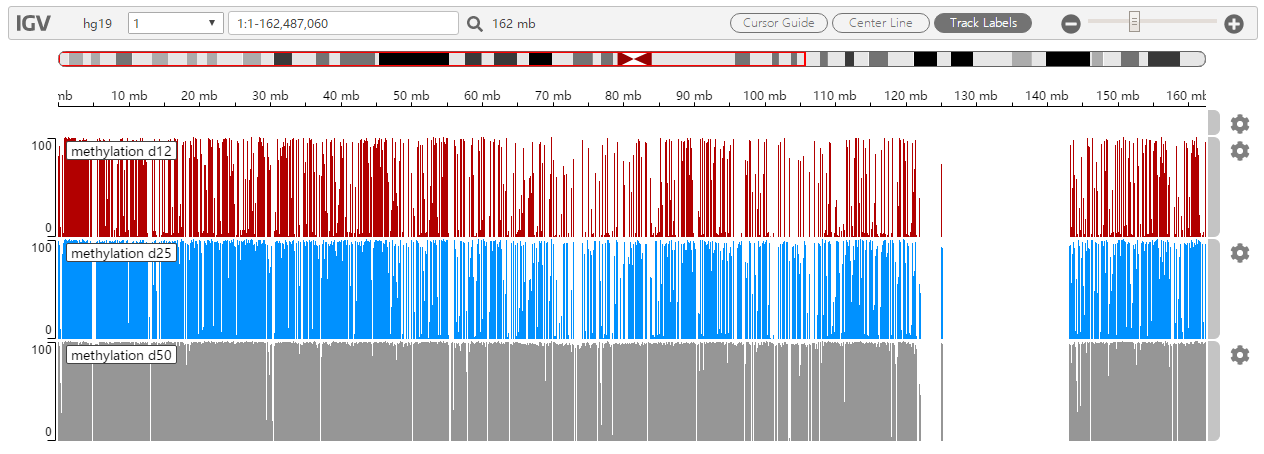


(D)

**Fig. 6** CGIs methylation analysis results. (A) The selection interface; (B) Distribution density of CGIs intersecting with different gene regions; (C) Distribution density of CGIs intersecting with gene body; (D) Mean methylation level of all CpG sites in CGIs.

# 3.2 GO enrichment analysis

After clicking the " GO enrichment analysis " link, users can use the selection interface (Fig. 7A) to choose the genome annotation version, maximum distance parameter, CGI density, and gene expression breadth classification. Users can obtain the GO enrichment analysis results by clicking the "Submit" button (Fig. 7A).

Each GO enrichment result consists of three charts: molecular function, cellular component, and biological process, which are represented by blue, orange, and green, respectively (Fig. 7B). GO charts show the most enriched GO annotation terms in the selected gene set. User can also click “Download detailed GO enrichment analysis results” button to download the detailed GO enrichment analysis results file (Fig. 7B)


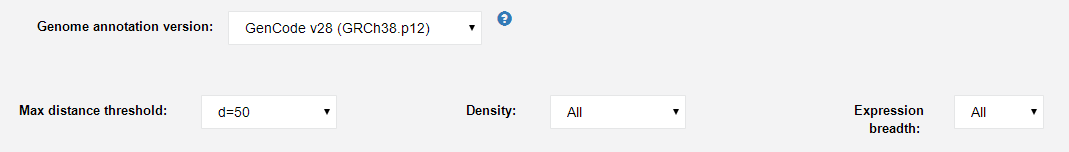


(A)


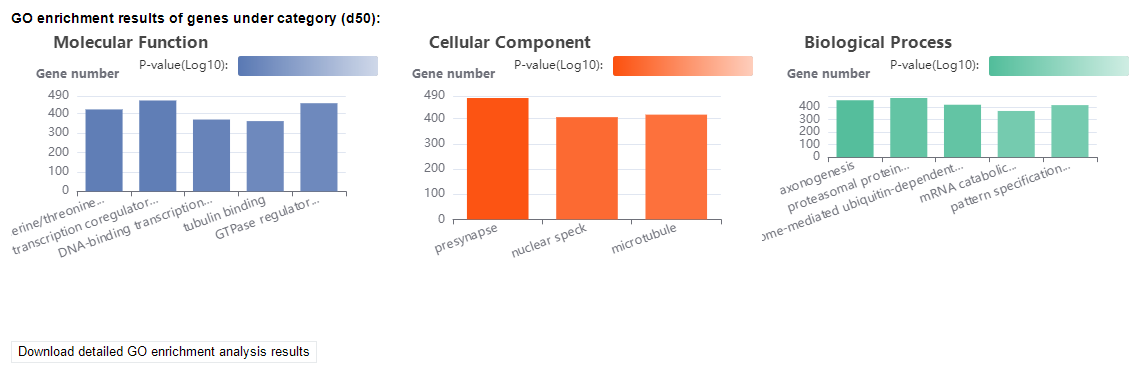


(B)

**Fig. 7** GO enrichment analysis. (A) The selection interface for gene annotation category. (B) The GO enrichment results of d=50 CGIs.
